# FOXM1-induced miR-552 expression contributes to pancreatic cancer progression by targeting multiple tumor suppressor genes

**DOI:** 10.7150/ijbs.56733

**Published:** 2021-03-01

**Authors:** Xiao Wang, Ning Dou, Jialin Wang, Yi Zhang, Yandong Li, Yong Gao

**Affiliations:** 1 Department of Oncology, Shanghai East Hospital, Tongji University School of Medicine, Shanghai 200120, China.; 2 Department of Oncology, Ren Ji Hospital, School of Medicine, Shanghai Jiao Tong University, Shanghai 200127, China.

**Keywords:** FOXM1, miR-552, pancreatic cancer, migration

## Abstract

Dysregulation of microRNAs (miRNAs) plays important roles during carcinogenesis. Forkhead box M1 (FOXM1), a well-known oncogenic transcription factor, has been implicated in the progression of multiple cancer types. To find out FOXM1-induced abnormal miRNAs in pancreatic cancer, we analyzed TCGA database and figured out miR-552 as the most relevant miRNA with FOXM1. Molecular experimental results demonstrated that FOXM1 transcriptionally activated miR-552 expression by directly binding to the promoter region of miR-552. In a pancreatic cancer tissue microarray, miR-552 expression was positively correlated with FOXM1 and high expression of miR-552 could predict poor patient outcome. Functionally, overexpression of miR-552 promoted pancreatic cancer cell migration and inhibition of miR-552 attenuated this phenotype. The inhibitory effect on cell migration caused by FOXM1 knockdown could be restored by exogenous expression of miR-552. By informatics analysis, we identified three tumor suppressor genes: DACH1, PCDH10 and SMAD4, all of which were negatively associated with FOXM1 and validated as functionally relevant targets of miR-552. Taken together, our findings provide a new FOXM1-miR-552-DACH1/PCDH10/SMAD4 axis to regulate pancreatic cancer cell progression and new opportunities for therapeutic intervention against this disease.

## Introduction

Pancreatic cancer is currently one of the most lethal diseases in the world 1. Pancreatic ductal adenocarcinoma (PDAC), which accounts for 90% of pancreatic cancer cases, is often used synonymously with pancreatic cancer and has an annually increasing incidence, especially in industrialized countries 2. Resection is the best choice for treatment of pancreatic cancer, but most patients have locally advanced or distant metastatic disease at the time of initial diagnosis due to the aggressive nature of this neoplasm 3. Although great efforts have been dedicated in the research of curative resection and adjuvant treatments, such as chemotherapy, immunotherapy and biological therapies, the prognosis of pancreatic cancer patients is far from satisfactory and the 5-year overall survival rate remains only 9% and median survival duration remains 6 months after diagnosis 4, 5. Therefore, further exploring the mechanisms underlying the pathogenesis of pancreatic cancer will help to find more potential therapeutic targets.

Forkhead box protein M1 (FOXM1), identified as a tumorigenic gene, has been implicated in several types of cancer including PDAC 6. FOXM1 belongs to the Forkhead box transcription factor superfamily, which shares an evolutionarily conserved 100 amino acid long winged-helix DNA-binding domain 7. Growing evidence has demonstrated that FOXM1 is frequently overexpressed in breast cancer, lung cancer, hepatocellular carcinoma, glioblastoma and pancreatic cancer. As an essential transcription regulator, it regulates the expression of many key genes and participates in multiple aspects of tumor progression, such as tumor cell survival, growth, angiogenesis, epithelial-to-mesenchymal transition (EMT) 8, 9. Recent reports have shown that FOXM1 contributes to pancreatic cancer invasion and progression by increasing the expression of MMP-9, MMP-2 and VEGF and downregulated FOXM1 expression results in the inhibition of pancreatic cancer cell growth and invasion 10. Moreover, FOXM1 promotes EMT and metastasis of pancreatic cancer cells via transcriptional regulation of the expression of uPAR as well as FOXM1-caveolin-1 signaling pathway 11, 12. So far many studies have revealed that microRNAs (miRNAs) are involved in the regulation of FOXM1 expression in cancer development 13. These miRNAs target and downregulate FOXM1, thereby leading to the inhibition of cancer cell growth and metastasis. However to date, the effects of FOXM1 on miRNA expression in pancreatic cancer development and progression remain to be clarified.

miRNAs are a class of endogenous and non-coding small RNAs that post-transcriptionally regulate gene expression 14. They are involved in a broad range of biological processes, such as proliferation, differentiation, stress response by mediating target mRNA degradation or translational repression 15, 16. Dysregulation of miRNAs is also associated with the initiation and progression of human cancers. For example, miR-200 family, miR-203 and miR-208 can trigger EMT to induce pancreatic cancer invasion and metastasis 17-19. Ectopic expression of miR-21 down-regulates PTEN and PDCD4, while miR-4935 targets GPC5 (Glypican 5), resulting in stimulation of Wnt/*β-*catenin signaling pathway to improve the metastasis of pancreatic cancer 20, 21. A series of miRNAs have been claimed to contribute to the process of pancreatic cancer progression. Nevertheless, the function and regulation of miR-552, which has been reported to enhance the invasion and metastatic capability in hepatocellular carcinoma, colorectal cancer and ovarian cancer, remain unknown in pancreatic cancer 22.

In this study, we aimed to explore FOXM1-regulated downstream miRNAs through analyzing the TCGA (The Cancer Genome Atlas) database and identified miR-552 as a new transcriptional target of FOXM1. We found that miR-552 was significantly upregulated and associated with poor prognosis in pancreatic cancer patients. This upregulation of miR-552 induced by FOXM1 further inhibited the expression of three downstream targets DACH1, PCDH10, SMAD4, which are known as tumor suppressors in pancreatic cancer progression.

## Materials and methods

### Tissue microarray

The human 293T cell line and pancreatic cancer tissue microarrays (Cat#: HPan-Ade180Sur-02) which contain 73 pairs of PDAC samples and adjacent normal tissues were obtained from Shanghai Outdo Biotech, China. Immunohistochemical staining for FOXM1 and *in situ* hybridization for miR-552 were respectively performed according to the commercial protocol (Outdo Biotech, Shanghai, China) using anti-FOXM1 antibody (Santa Cruz Biotechnology, USA) or 3' and 5' DIG labeled LNA miR-552 probes (Ambion, USA). All the samples were identified by two experienced pathologists independently blinded to the clinical data. The following expression levels were based on the score obtained by the intensity and percentage of the positive staining. The intensity was recorded as 0, 1, 2, and 3 referring to negative, weak, moderate, and strong staining, respectively. Grade 0, 1, 2 group were classified as 'low expression' while grade 3 group were classified as 'high expression'. The research on the use of human tissue samples was approved by the Medical Ethics Committees of Shanghai East Hospital, Tongji University.

### Cell culture

The human pancreatic adenocarcinoma cell lines PANC1, AsPC1 and SW1990 were purchased from Shanghai Cell Bank of Chinese Academy of Sciences. All the cell lines were cultured in Dulbecco's Modified Eagle's medium (DMEM) supplemented with 10% fetal bovine serum (Gibco, USA), 100 U/mL penicillin (Gibco, USA) and 100 mg/mL streptomycin (Gibco, USA) at 37 °C in a humidified atmosphere containing 5% CO_2_.

### RNA isolation and quantitative real-time PCR (qRT-PCR)

Total RNA was extracted from cells using TRIzol (Invitrogen, USA) according to the manufacturer's protocol. RNA (1μg) was reverse-transcribed using a TaKaRa PrimeScript RT reagent kit (Takara, Japan). Quantitative real-time PCR was performed using SYBR green Supermix (Applied Biosystems, USA) in an ABI 7500 PCR system (Applied Biosystems, USA). Each reaction was performed in triplicate. Primer sequences are listed in Supplementary Table 1. The mRNA levels were normalized by GAPDH.

### Transient transfection and stable cell line establishment

The miR-552-3p mimics/mimic negative control and miR-552-3p inhibitors/inhibitor negative control were purchased from GenePharma, Shanghai, China. The specific inhibitor of miR-552-3p was single strand of antisense of miR-552-3p with 2′-O-methyl modification. Its working concentration was 100 nM. Small inference RNAs (siRNAs) against DACH1, PCDH10, SMAD4 and control (siNC) were also chemically synthesized by GenePharma, Shanghai. The sense sequences for each gene as follows: si-DACH1, 5'-GGCAGCUUCAACAGAUAGUdTdT-3'; si-PCDH10, 5'-GCUCCAAUGUACCCAGUAAdTdT-3'; si-SMAD4, 5'-CAGAUUGUCUUGCAACUUCAG-3'; si-NC: 5'- UUCUCCGAACGUGUCACGUdTdT-3'. The FOXM1 expression plasmid (pcDNA3.1-FOXM1) is a gift from Dr. Keping Xie, MD Anderson Cancer Center, USA. Transient cell transfection with above mimics, inhibitors, siRNAs or plasmids were conducted using Lipofectamine 3000 (Invitrogen, USA) in line with the manufacturer's instructions. The cells stably silencing FOXM1 expression were generated through transducing LV-shFOXM1 lentiviral particles followed by puromycin selection as previously described 23. The lentivirus expressing miR-552 precursor (pre-miR-552) was purchased from Shanghai Tuzhu Corporation, China.

### Chromatin immunoprecipitation assay (ChIP)

PANC1 cells (1×10^7^) were prepared for ChIP assay. ChIP experiments were performed with chromatin immunoprecipitation assay kit (Millipore, CA, USA) according to the manufacturer's instructions. Briefly, cell sediments were crosslinked with 1% formaldehyde for 15 mins. Crosslinked chromatin was sonicated and incubated with 5 μg anti-FOXM1 antibody (Santa Cruz Biotechnology, USA) at 4 °C overnight. Co-precipitated DNA was analyzed using PCR to amplify a 133-bp region of pri-miR-552 promoter and PCR products were resolved electrophoretically on a 2% agarose gel. Also, the immunoprecipitated DNA was quantitated by real-time PCR to measure FOXM1 binding levels, which were normalized to 2% input. Isotype-matched IgG (5 μg) was used as a negative control. The 133-bp promoter sequence was amplified with the following primer pairs: Forward, 5'-TGCCTGTTGGTTGAAGATG-3'; Reverse, 5'-CCAAAGATTCATCCAAGTTG-3'.

### Luciferase reporter assay

Dual-luciferase reporter assay was used to evaluate the direct effect of FOXM1 on miR-552 promoter and miR-552's direct downstream target DACH1, PCDH10, SMAD4. For promoter activity assay, the promoter of pri-miR-552 (-542 to +208, the first nucleotide of pre-miR-552 is defined +1) was inserted into the pGL3-Basic plasmid, and the 3'-UTR of DACH1, PCDH10, and SMAD4 were cloned and inserted into pGL3-control vector, respectively. Pancreatic cancer cells were co-transfected with above reporter constructs, an internal control (pRL-SV40) and corresponding FOXM1 expression plasmid or miR-552 mimics/inhibitors. After transfection for 24 h, the cells were harvested and lysed with 100 μl passive lysis buffer, luciferase activities in the cell lysates were measured using a dual-luciferase reporter assay system (Promega, USA). The data presented were obtained from three independent experiments.

### Western blotting

Cells were lysed in in RIPA Lysis Buffer (Beyotime Biotechnology, China), and the protein concentration was determined by a Pierce™ BCA Protein Assay Kit (Thermo Fisher Scientific, USA). The proteins were separated by 10% sodium dodecyl sulfate polyacrylamide gel electrophoresis (SDS-PAGE) for approximately 1.5 h at 90 V-120 V, and then transferred to PVDF membranes (Millipore, USA) at 250 mA for 60 min. After blocked with 5% non-fat milk, the membranes were incubated overnight at 4 °C with specific primary antibodies (FOXM1, 1:200, Santa Cruz Biotechnology, USA; PCDH10, 1:500, Proteintech, China; DACH1, 1:500, Proteintech, China; SMAD4, 1:500, Proteintech, China; β-actin, 1:2000, Proteintech, China). After being washed with TBST (Tris-buffered saline plus Tween-20) three times, the membranes were incubated with corresponding secondary antibodies for 1 h. Bound antibodies were visualized with Li-Cor Odyssey imaging system (Li-COR Biosciences, USA) according to the manufacturer's instructions.

### Cell migration assay

Transwell assay and wound-healing assay were conducted to assess the pancreatic cancer cells invasive ability. Cancer cells were seeded into top chamber with 400 μl serum-free medium. The lower chamber was filled with 800 μl culture medium containing 10% FBS as a chemo-attractant. After 24 h of incubation, the non-migrating cells were scraped off by cotton swabs and the migrated cells were fixed with 4% paraformaldehyde, stained with 0.1% crystal violet and then photographed and counted under a microscope. For the wound-healing assay, after the cells grew in 90-95% confluence in 6-well plates, the wound closure was generated by scratching the surface of the plates with a 200-μl pipette tip. Then the cells were incubated in DMEM medium without FBS. The wound closure was photographed at indicated time points. Experiments were performed at least in triplicate.

### TCGA datasets analysis

PDAC patients' TCGA data on FOXM1 RNA expression (96 samples) and miRNA-Seq profiling were downloaded from the Cancer Genomics Brower of the University of California, Santa Cruz (UCSC; https://genome-cancer.ucsc. edu/). There are 96 common samples in total with both FOXM1 mRNA and miRNA expression. Then we identify the most relevant miRNA from 508 miRNAs with FOXM1 according to miRNA expression level.

### *In vivo* metastases assay

Four-week-old BALB/c nude mice were purchased from Sippr-BK laboratory animal corporation, Shanghai, China. 2×10^6^ of AsPC1 cells with stably expressing pre-miR-552 or silencing FOXM1 were injected via the lateral tail vein. Mice were sacrificed and their lungs were removed at 6 weeks after injection. Lungs from the mice of all groups were photographed and fixed in formalin. After being immersed in an ascending series of alcohols, and embedded in paraffin, tissue sections were cut and routinely stained with H&E to assess the metastases. All animal experiments were performed under approval by the Ethics Committee of Shanghai East Hospital.

### Statistical analysis

Each experiment was carried out independently at least three times with similar findings; results from one representative experiment are presented. Statistical analyses were performed using* χ*^2^ test or the Student's *t* test (two-tailed unpaired) by GraphPad Prism 7. All the data are presented as the mean ± SD. Survival curves were assessed with the Kaplan-Meier method and compared by the log-rank test. The significance of the patient specimen data was determined using the Pearson correlation test. *p* values less than 0.05 were considered statistically significant. *p < 0.05, **p < 0.01, ***p < 0.001.

## Results

### Identification of miR-552 as a direct transcriptional target of FOXM1 in pancreatic cancer cells

**Figure 1:**
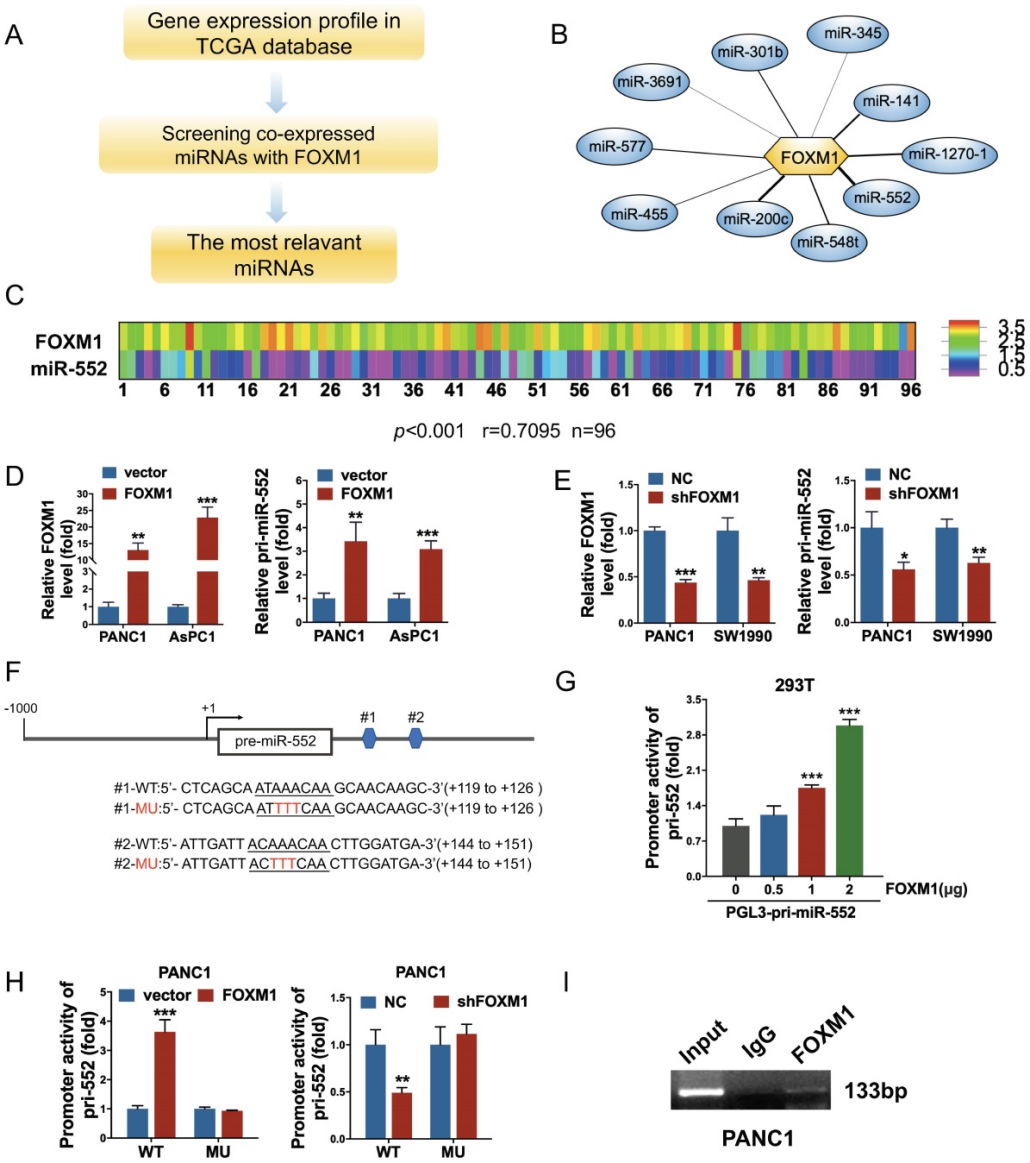
** FOXM1 regulated miR-552 expression in pancreatic cancer cells. (A)** The flow chart showed how to find the FOXM1-related miRNAs. **(B)** miR-552 is the most relevant miRNA with FOXM1. **(C)** FOXM1 mRNA expression positively correlated with miR-552 in 96 pairs of PDAC patients using linear regression models. (*p*<0.001 r=0.7095 n=96). **(D)** qRT-PCR analysis of FOXM1 and pri-miR-552 expression in PANC1 and AsPC1 cells transfected with FOXM1 or control vector. **(E)** qRT-PCR analysis of FOXM1 and pri-miR-552 expression in PANC1 and SW1990 cells transfected with shFOXM1 and a control vector. **(F)** Representative scheme of wild type and mutant FOXM1-binding site in pri-miR-552 (nucleotides +119 to +126 and +144 to +151). **(G)** Luciferase activity of pri-miR-552 promoter in 293T cells after transfection with different concentration of FOXM1 plasmid. **(H)** Luciferase activity of wild type and mutant pri-miR-552 promoter in PANC1 cells when FOXM1 was overexpressed or knocked down. **(I)** FOXM1 directly bound to the promoter region of pri-miR-552. ChIP assay was performed in PANC1 cells and anti-FOXM1 antibody and normal IgG were subjected to the cell lysates, and 1% of the total cell lysates was used for PCR before immunoprecipitation (input control).

To explore FOXM1-regulated miRNAs, we analyzed the expression of FOXM1 and all of miRNAs in cancer patients from the PDAC dataset of TCGA following the steps in Figure 1A. The correlation index between FOXM1 and miRNAs was obtained using Pearson's correlation analysis. A series of miRNAs were screened out as presented in Figure 1B and Supplementary Table 2. Among them, miR-552 was the most relevant miRNA with FOXM1 (r=0.7095, *p*<0.001) in 96 PDAC tissues of this database (Figure 1C). To examine if FOXM1 regulates miR-552 expression at transcriptional level, we first investigated the expression of pri-miR-552 (primary miR-552) in FOXM1 overexpression cells or knockdown cells by qRT-PCR. As shown in Figure 1D and Figure 1E, pri-miR-552 expression was increased with FOXM1 upregulation and decreased with FOXM1 downregulation, indicating that miR-552 expression was regulated by FOXM1. Furthermore, we found two putative binding sites of FOXM1 nearby miR-552 precursor region (Figure 1F). To verify if the binding elements affect the transcription of pri-miR-552 by FOXM1, we generated a luciferase reporter construct which contains the two binding sequences and the upstream promoter of miR-552. The data from dual luciferase assays demonstrated that the promoter activity of miR-552 could be activated by FOXM1 in a dose-dependent manner (Figure 1G). Mutation of the binding sites impaired the effect of FOXM1 on miR-552 promoter activities (Figure 1F and Figure 1H). In addition, the results of ChIP assay showed that FOXM1 could bind to the promoter region of miR-552 (Figure 1I). These findings above strongly suggest that FOXM1 directly regulates miR-552 expression at transcriptional level.

### The expression of miR-552 is associated with FOXM1 and predicts poor prognosis

**Figure 2:**
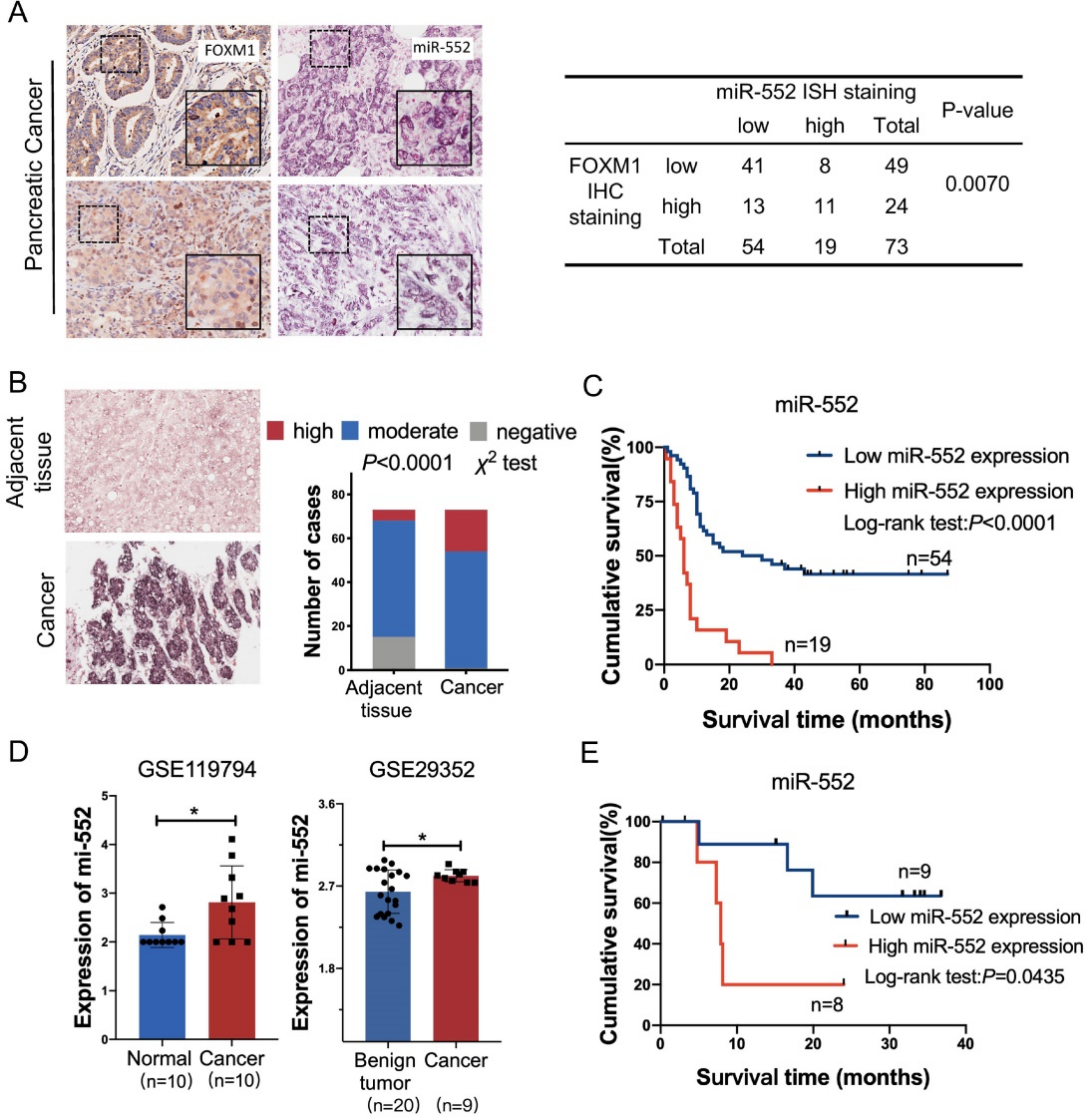
** The correlation of miR-552 with FOXM1 expression in pancreatic cancer tissues. (A)** Statistical analysis of immunohistochemical staining of FOXM1 and *in situ* hybridization of miR-552 in human PDAC specimens. Representative images of FOXM1 and miR-552 were shown. **(B)** miR-552 *in situ* hybridization analysis and representative images in cancer tissue and adjacent normal pancreatic tissue were shown. Data are analyzed by χ^2^ test. **(C)** Kaplan-Meier curve for overall survival of above PDAC patients. These patients were stratified as high and low according to the miR-552 expression signature in primary tumors of TMAs (log-rank test), *p*<0.0001. **(D)** The expression of miR-552 from GEO database was higher in cancer tissues than non-cancerous tissues. **(E)** Association of miR-552 expression with Overall Survival (OS) rate in patients with PDAC. Patients with high miR-552 expression had much shorter OS durations than did patients with low miR-552 expression (log-rank test),* p*=0.0435. All the PDAC patients in (E) were elaborated from http://www.kmplot.com.

To elucidate the correlation between miR-552 and FOXM1 in clinical samples, we detected the expression of FOXM1 and miR-552 by immunohistochemical staining and *in situ* hybridization (ISH) in a pancreatic cancer tissue microarray, respectively. The results indicated that miR-552 was closely associated with FOXM1 expression in the 73 cancer tissues (Figure 2A). In the meantime, miR-552 expression was significantly higher in the cancer tissues compared with their corresponding adjacent non-cancerous tissues (Figure 2B) and the patients with high expression of miR-552 had shorter overall survival time (Figure 2C). Another result from GEO datasets also showed that miR-552 was upregulated in pancreatic cancer tissues in contrast with non-cancerous tissues (Figure 2D). The indicator role of miR-552 for patients' prognosis was confirmed by the data from Kaplan-Meier Plotter cohort (http://www.kmplot.com) (Figure 2E). These results indicate that miR-552 may play critical roles in pancreatic cancer development and its expression could be a valuable biomarker for this disease.

### miR-552 facilitates cell migration in pancreatic cancer cells

**Figure 3:**
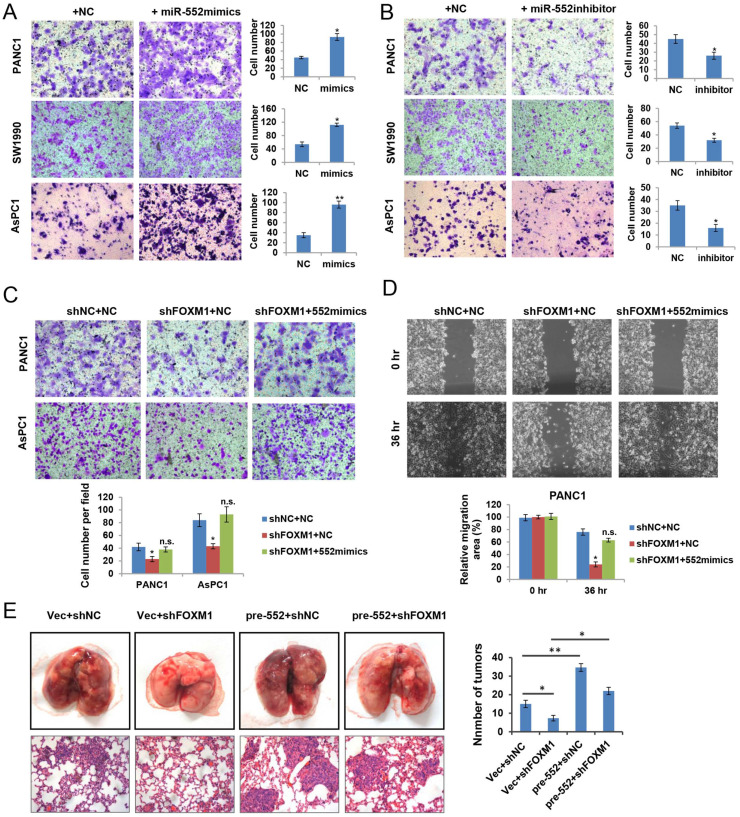
** miR-552 promoted pancreatic cancer cell migration. (A)** Overexpression of miR-552 enhanced the abilities of migration in PANC1, SW1990 and AsPC1 cells. **(B)** Inhibition of miR-552 by its specific inhibitors reduced the migration ability of PANC1, SW1990 and AsPC1 cells. **(C)** Overexpression of miR-552 restored the phenotypic defect caused by FOXM1 knockdown in PANC1 and AsPC1 cells by Transwell assay. **(D)** Overexpression of miR-552 restored the phenotypic defect caused by FOXM1 knockdown in PANC1 cells by wound healing assay. **(E)** Lung metastasis assay was performed by tail vein injection of four groups of AsPC1 cells as indicated. The representative gross lungs with metastases and hematoxylin and eosin (HE) stained lung sections in each group (n=5) were shown. *p <0.05, **p <0.01, n.s., not statistically significant.

To ascertain the biological functions of miR‑552 in pancreatic cancer, we firstly examined the effect of miR-552 expression on cell growth. Unexpectedly, no obvious phenotype was observed when overexpression of miR-552 by transfecting miR-552 mimics into pancreatic cancer cells (data not shown). Next, we attempted to investigate whether miR-552 could affect the migration ability of pancreatic cancer cells, which were important aspects for metastasis. As shown in Figure 3A, miR-552 mimics significantly enhanced the migration of PANC1, SW1990 and AsPC1 cells by transwell assays. Conversely, silencing miR-552 expression via its specific inhibitors markedly reduced the migration in the same three cell lines (Figure 3B). To further understand the role of miR-552 in FOXM1-mediated pancreatic cancer cell migration, we introduced miR-552 mimics into PANC1 and AsPC1 cells with FOXM1 knockdown. The resulting data showed that miR-552 overexpression rescued the defects of FOXM1 knockdown on cell migration in both transwell assay and wound healing assay (Figure 3C and Figure 3D). Similarly, results from the pulmonary metastasis model in nude mice indicated that miR-552 could promote cell metastasis *in vivo* and restore the defect of FOXM1 knockdown consistent with the *in vitro* findings (Figure 3E). Taken together, these results suggest that miR-552 positively regulates pancreatic cancer cell migration.

### DACH1, PCDH10 and SMAD4 act as direct downstream targets of miR-552

**Figure 4:**
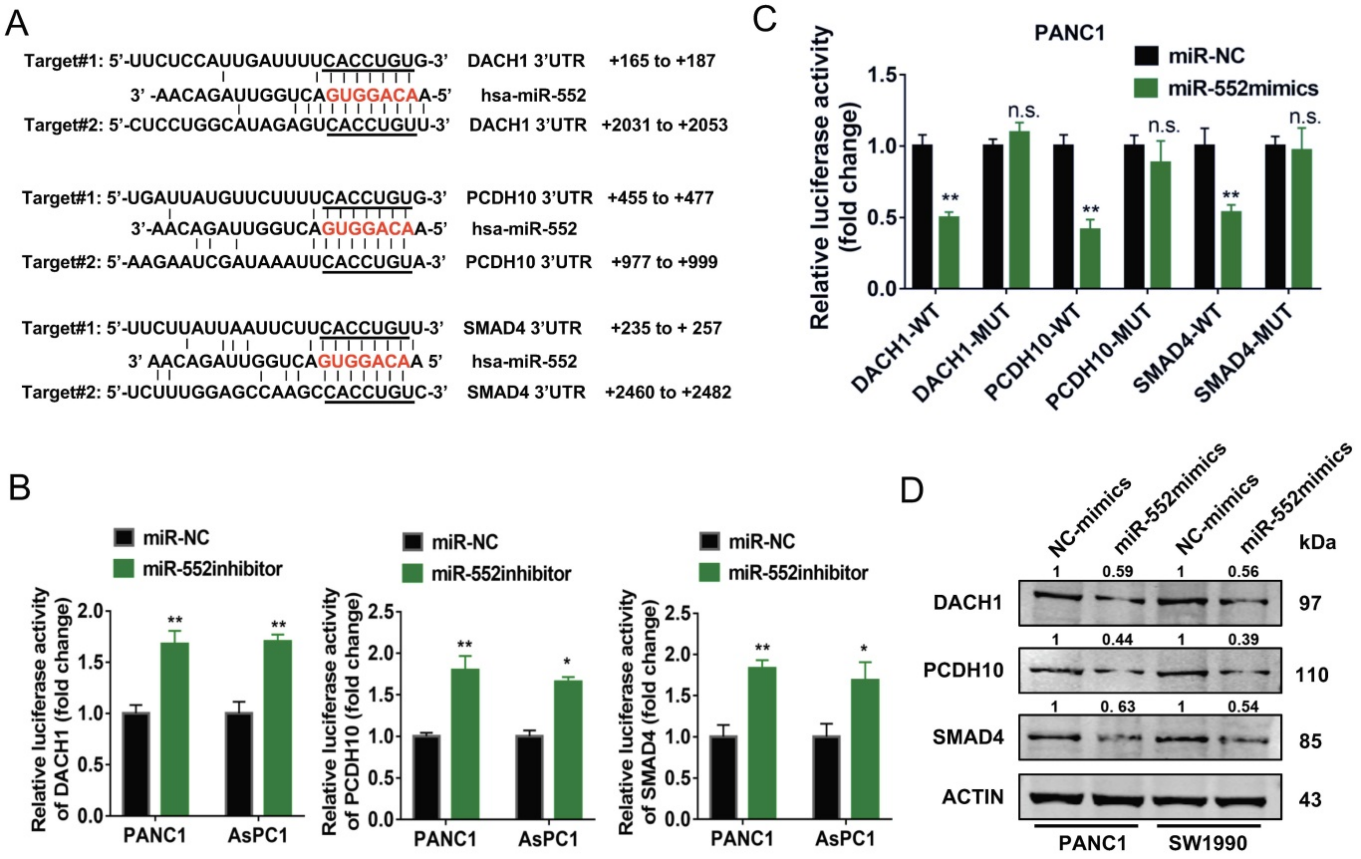
** miR-552 repressed the expression of DACH1, PCDH10 and SMAD4. (A)** Schematic wild type and mutant binding site of miR-552 in DACH1, PCDH10, and SMAD4. **(B)** The dual luciferase reporter assays revealed that luciferase activities was increased in PANC1 and AsPC1 cells after co-transfection with miR-552 inhibitor and wild-type 3'-UTR reporters of DACH1, PCDH10 or SMAD4. **(C)** Dual luciferase reporter assay in PANC1 cells co-transfected with miR-552 mimics and mutant type or wild type 3'-UTR reporters of DACH1, PCDH10 and SMAD4. **(D)** Western blotting showed that DACH1, PCDH10 and SMAD4 were downregulated in PANC1 and SW1990 cells after transfection of miR-552 mimics. *p <0.05, **p <0.01.

It is known to us that miRNAs act as inhibitors of target genes to produce their function. By TargetScan prediction tool we found several target genes of miR-552 which function as tumor suppressors in tumors. Among them, we selected three genes DACH1, PCDH10, and SMAD4, the expression of which were negatively correlated with FOXM1 from online analyses in cBioPortal database (https://www.cbioportal.org) (Figure 4A and Supplementary Figure 1). To examine whether miR-552 is able to downregulate the expression of these three genes in pancreatic cancer cells, a dual luciferase reporter assay was performed. The results indicated that relative luciferase activities of 3′-UTR sequence of DACH1, PCDH10, or SMAD4 were de-repressed by introduction of miR-552 inhibitors in PANC1 and AsPC1 cells (Figure 4B). On the other hand, overexpression of miR-552 significantly decreased the relative luciferase activities of the 3′-UTR of the three genes, but had no evident influence on the 3′-UTR of the three genes with miR-552 binding site mutations (Figure 4C), indicating that miR-552 may repress the expression of these genes via their 3′-UTR sequence. Furthermore, western blot results showed that the protein levels of DACH1, PCDH10, and SMAD4 were reduced in PANC1 and SW1990 cell lines upon transfection with miR-552 mimics (Figure 4D). These collective results imply that DACH1, PCDH10 and SMAD4 may be direct targets of miR-552.

### FOXM1 inhibits the expression of DACH1, PCDH10 and SMAD4 via miR-552

**Figure 5:**
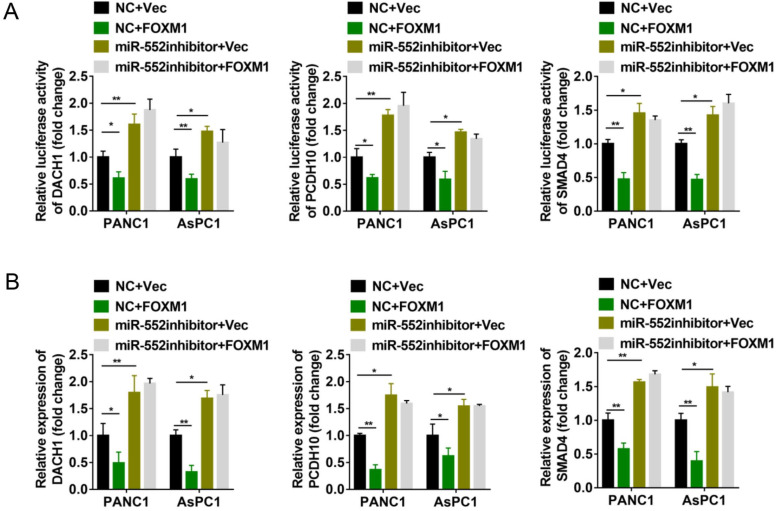
** miR-552 was required for the regulation of DACH1, PCDH10 and SMAD4 by FOXM1. (A)** Luciferase reporter assays were performed to detect the 3'-UTR activity of DACH1, PCDH10 and SMAD4 in PANC1 and AsPC1 cells, respectively. **(B)** The mRNA expression level of DACH1, PCDH10 and SMAD4 in PANC1 and AsPC1 cells were detected via qRT-PCR. The cells in (A) and (B) were divided into four groups transfected with NC+Vec, NC+FOXM1, miR-552 inhibitor+Vec, miR-552 inhibitor+FOXM1 as indicated. *p <0.05, **p <0.01.

Since miR-552 is regulated by FOXM1, we next explored whether FOXM1 could regulate the expression of DACH1, PCDH10 and SMAD4. The dual-luciferase reporter assays revealed that transient overexpression of FOXM1 decreased the relative luciferase activity of 3′-UTR of the three genes, whereas miR-552 silencing by its inhibitors destroyed the effects caused by FOXM1 overexpression (Figure 5A). To further confirm this result, the mRNA level of DACH1, PCDH10 and SMAD4 were detected by qRT-PCR. In accordance with dual-luciferase reporter assays, FOXM1 exhibited the suppressive effect on the expression of these three genes, and this suppression was shown in a miR-552-dependent manner (Figure 5B). Thus, these results demonstrate that FOXM1/miR-552 axis inhibits the expression of three tumor suppressor genes DACH1, PCDH10 and SMAD4 at mRNA level.

### DACH1, PCDH10 and SMAD4 function as downstream effectors of FOXM1/miR-552 axis in pancreatic cancer cell migration

**Figure 6:**
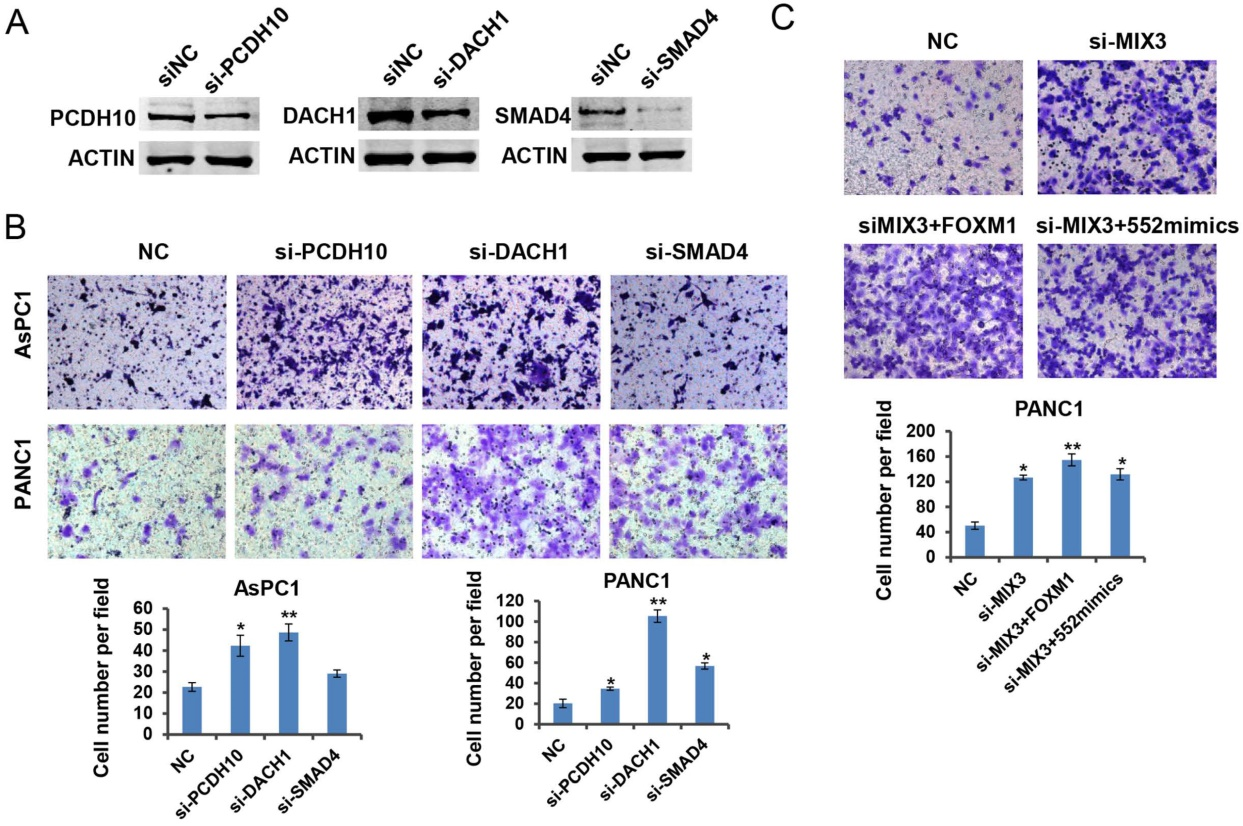
** PCDH10, DACH1, and SMAD4 may function as tumor suppressors downstream of FOXM1/miR-552 axis. (A)** PCDH10, DACH1 and SMAD4 protein expression were detected by western blotting after transfection with si-PCDH10, si-DACH1 or si-SMAD4 in PANC1 cells. **(B)** PCDH10, DACH1 or SMAD4 knockdown significantly increased cell migration ability in transwell chamber assays. **(C)** The effects of FOXM1 or miR-552 overexpression on migration in PANC1 cells with combined knockdown of PCDH10, DACH1 and SMAD4. *p <0.05, **p <0.01.

Previous reports have shown the tumor suppressive roles of DACH1, PCDH10 and SMAD4 in tumorigenesis 24-26. To verify the roles of the three genes in pancreatic cancer, we downregulated their expression by siRNA transfection in PANC1 cells, respectively. Western blot results indicated the interference efficiency for each specific siRNA (Figure 6A). As expected, downregulation of each gene indicated an enhanced migration capacity except the phenotype caused by SMAD4 knockdown in AsPC1 cells (Figure 6B). It might be attributed to the deficiency of SMAD4 protein in AsPC1 cells. To assess whether the three tumor suppressor genes are involved in FOXM1/miR-552 axis induced cell migration, we transfected a mixture of three siRNAs into PANC1 cells, and observed a strong migration phenotype relative to the control group. Ectopic expression of FOXM1 or miR-552 did not show an enhanced migration phenotype in these cells (Figure 6C), suggesting that DACH1, PCDH10 and SMAD4 mediate the function of FOXM/miR-552 axis in cell migration.

## Discussion

In the present study, we identified miR-552 as one of new target genes that induced by FOXM1 through bioinformatics analysis and molecular experiments. We found FOXM1 transcriptionally activated miR-552 expression and miR-552 is positively associated with pancreatic cancer metastasis. Subsequently, we further determined the roles of miR-552 in pancreatic cancer cells. Through gain-of-function and loss-of-function assays, we demonstrated that miR-552 could act as an oncogenic miRNA in promoting pancreatic cancer cell migration and metastasis, in line with its clinical significance.

FOXM1 is a well-documented transcriptional activator with potential oncogenic property. It has been reported that many downstream effectors involved in tumor development and progression were regulated by FOXM1 at transcriptional level, such as the encoding genes, MMP9, MMP2 and uPA/uPAR 10, 12. On the other hand, several miRNAs, including miR-370, miR-200b and miR-494 have been identified as negative regulators of FOXM1 at post-transcriptional level 13, 27. Nevertheless, little is known whether FOXM1 transcriptionally activates the expression of miRNAs in cancer. To this end, we analyzed the expression of FOXM1 and all miRNAs in public TCGA database and found miR-552, which could be the most relevant miRNA with FOXM1 in pancreatic cancer. Results from ChIP assays and dual-luciferase assays indicated that FOXM1 binds to miR-552 promoter to induce its expression. Meanwhile, the positive correlation between FOXM1 and miR-552 was verified by IHC and ISH staining in a pancreatic cancer tissue microarray. Functionally, we observed that miR-552 is required to maintain the phenotypes of FOXM1-induced enhanced cell migration *in vitro* and *in vivo*. Thus, our findings for the first time suggest that miR-552 is a new downstream functional target of FOXM1.

miR-552 is located in human chromosome 1p34.3 and frequently overexpressed in different malignancies. Previous studies have demonstrated that miR-552 functions as a potential oncogene in a series of cancer types and figure out several downstream targets, such as AJAP1 in liver cancer, Smad2 and DACH1 in colorectal cancer, TIMP2 and WIF1 in osteosarcoma 28-32. In our study, we identified three tumor suppressor genes, DACH1, PCDH10 and SMAD4 as targets of miR-552 in pancreatic cancer. DACH1 is a paralog of Drosophila dachshund (dac) gene in vertebra, which is expressed widely in normal adult tissues, and loss of DACH1 expression predicts poor outcome in many cancers 33. PCDH10 belongs to the non-clustered protocadherin protein family encoding calcium-dependent adhesion protein and is known as a tumor suppressor gene in many tumors. It has been reported that high methylation level of PCDH10 predicts poor prognosis in patients with PDAC 25. SMAD4 is an important tumor suppressor whose mutation occurs in around 50% of pancreatic cancers. Patients with SMAD4 gene inactivation might be spared the risk of surgery because their cancer is more likely to metastasize, whereas patients with intact SMAD4 may benefit from the local control provided by neoadjuvant therapy and surgical resection 26. It is worth noting that the cell fate determination factor, DACH1 shares the DNA binding sequence at a high similarity with FOXM1 and could displace FOXM1 from the promoter of the targeted genes to repress FOXM1 target gene expression 24, 34. Interestingly, herein FOXM1 is an upstream effector of miR-552 and is negatively correlated with DACH1. Therefore, further work should be performed to investigate whether DACH1 could affect miR-552 expression by competitive binding to the miR-552 promoter with FOXM1.

In summary, this study provides mechanistic evidence for a tumorigenic role of miR-552 in pancreatic cancer and uncovers a novel FOXM1-miR-552-DACH1/PCDH10/SMAD4 axis that promotes pancreatic cancer progression. Our findings offer a new insight into the mechanism underlying the progression and metastasis of pancreatic cancer, which helps to explore novel targets for prognosis, prevention and therapy of this disease.

## Supplementary Material

Supplementary figures and tables.
