# Supplementary material for: FOXM1-induced miR-552 expression contributes to pancreatic cancer progression by targeting multiple tumor suppressor genes
Source: Int J Biol Sci. 2021 Mar 1;17(4):915–25. doi: 10.7150/ijbs.56733 (PMC8040302; doi:10.7150/ijbs.56733)

Supplementary Table 1 Primers for qRT-PCR used in this study

	Forward Primer	Reverse Primer
GAPDH	AAGGTCGGAGTCAACGGATTT	CCATGGGTGGAATCATATTGG
pri-miR-552	TAGCCAAGTGTTCACAGCAG	TGCTGAGTAGTATTCCATCCT
DACH1	GGAATGGATTGTGGCTGAAC	GGTATTGGACTGGTACATCAAG
PCDH10	ACTGCTATCAGGTATGCCTG	GTCTGTCAACTAGATAGCTG
SMAD4	ACGAACGAGTTGTATCACCTGG	TGCACGATTACTTGGTGGATG
FOXM1	GACTTCTTGGGTCTTGGGGTG	GGAGGAAATGCCACACTTAGCG

Supplementary Table 2 The correlation index between FOXM1 and miRNAs was obtained from TCGA using Pearson's correlation analysis. 20 miRNAs with r>0.3 or r<-0.3 and *p*<0.05 were listed.

Gene	miRNA	COR	*P*-Value
FOXM1	hsa-mir-552	0.709504979	4.44E-16
FOXM1	hsa-mir-200c	0.59424544	1.74E-10
FOXM1	hsa-mir-141	0.57463921	9.23E-10
FOXM1	hsa-mir-1270-1	0.409404234	3.45E-05
FOXM1	hsa-mir-548t	0.391259527	8.09E-05
FOXM1	hsa-mir-301b	0.337495172	0.000772
FOXM1	hsa-mir-577	0.3352254	0.000842
FOXM1	hsa-mir-455	0.331743409	0.00096
FOXM1	hsa-mir-345	0.329564381	0.001042
FOXM1	hsa-mir-3691	0.316000111	0.00171
FOXM1	hsa-mir-3934	0.301879167	0.002798
FOXM1	hsa-mir-26a-1	-0.300833896	0.002899
FOXM1	hsa-mir-195	-0.30230183	0.002758
FOXM1	hsa-mir-130a	-0.312276676	0.001952
FOXM1	hsa-mir-664	-0.313538954	0.001867
FOXM1	hsa-mir-338	-0.321394993	0.001408
FOXM1	hsa-mir-218-1	-0.321838801	0.001386
FOXM1	hsa-mir-146b	-0.322973744	0.00133
FOXM1	hsa-mir-218-2	-0.323900591	0.001285
FOXM1	hsa-mir-497	-0.333376179	0.000903

Supplementary Figure 1. The association of FOXM1 with DACH1, PCDH10 and SMAD4.

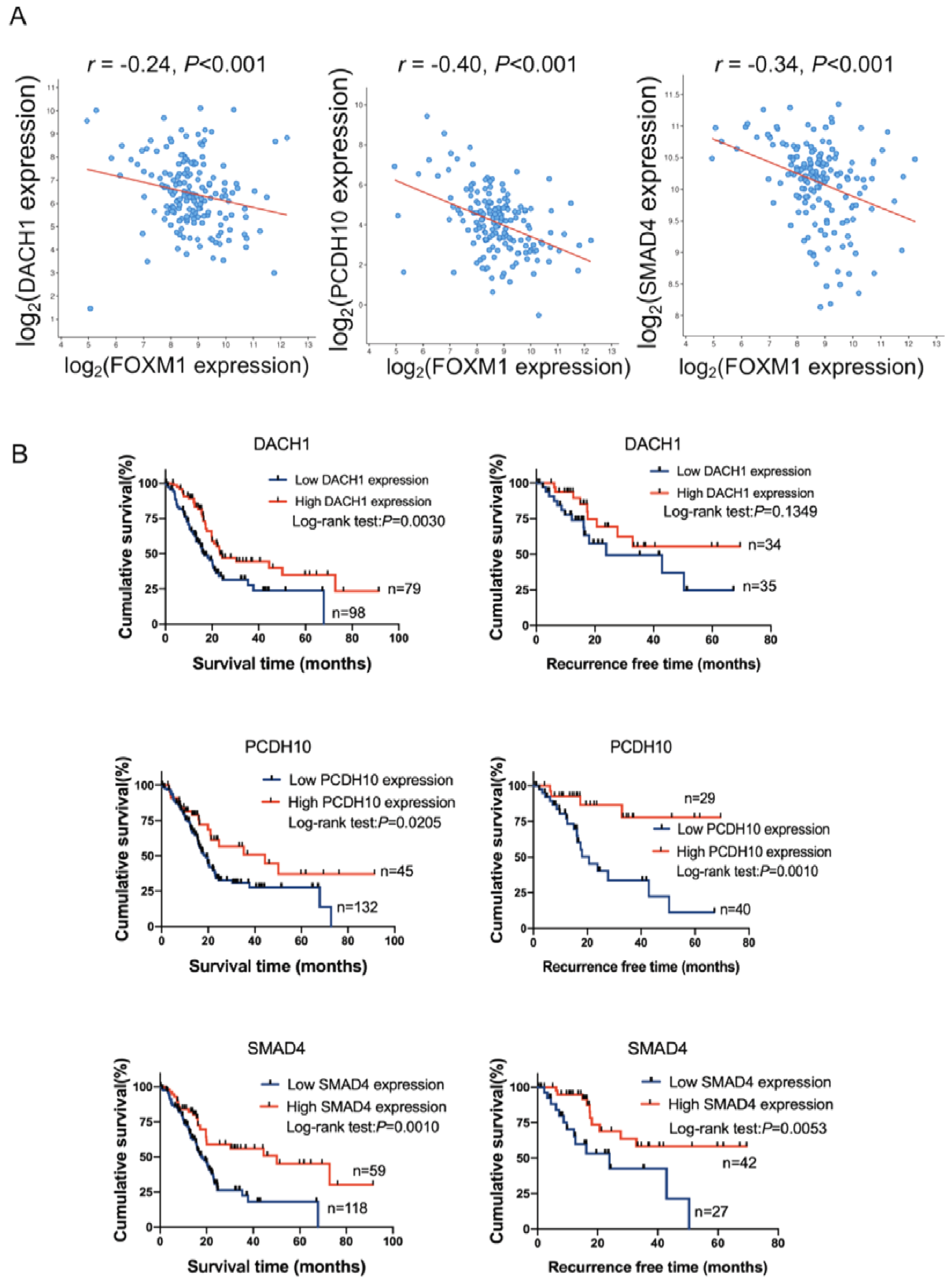

Supplement: Supplementary file 1 — Supplementary figures and tables. [file ijbsv17p0915s1.pdf]
